# Comprehensive Analysis of the Tumor Microenvironment and Ferroptosis-Related Genes Predict Prognosis with Ovarian Cancer

**DOI:** 10.3389/fgene.2021.774400

**Published:** 2021-11-15

**Authors:** Xiao-xue Li, Li Xiong, Yu Wen, Zi-jian Zhang

**Affiliations:** Department of General Surgery, The Second Xiangya Hospital of Central South University, Changsha, China

**Keywords:** ovarian cancer, tumor infiltrating immune cells, ferroptosis, prognostic, the cancer genome atlas

## Abstract

The early diagnosis of ovarian cancer (OC) is critical to improve the prognosis and prevent recurrence of patients. Nevertheless, there is still a lack of factors which can accurately predict it. In this study, we focused on the interaction of immune infiltration and ferroptosis and selected the ESTIMATE algorithm and 15 ferroptosis-related genes (FRGs) to construct a novel E-FRG scoring model for predicting overall survival of OC patients. The gene expression and corresponding clinical characteristics were obtained from the TCGA dataset (n = 375), GSE18520 (n = 53), and GSE32062 (n = 260). A total of 15 FRGs derived from FerrDb with the immune score and stromal score were identified in the prognostic model by using least absolute shrinkage and selection operator (LASSO)–penalized COX regression analysis. The Kaplan–Meier survival analysis and time-dependent ROC curves performed a powerful prognostic ability of the E-FRG model via multi-validation. Gene Set Enrichment Analysis and Gene Set Variation Analysis elucidate multiple potential pathways between the high and low E-FRG score group. Finally, the proteins of different genes in the model were verified in drug-resistant and non–drug-resistant tumor tissues. The results of this research provide new prospects in the role of immune infiltration and ferroptosis as a helpful tool to predict the outcome of OC patients.

## Introduction

Ovarian cancer (OC) is one of the most serious gynecological diseases and the second most common gynecological disease that causes female deaths worldwide, seriously endangering women’s health and safety (BRAY et al., 2018). Surgery combined with chemotherapy and targeted immunotherapy have greatly improved the survival rate of OC patients in recent times (CORRADO et al., 2019; LI et al., 2019). However, the survival rate of OC has not changed even in developed countries, such as the United States (GIAMPAOLINO et al., 2019). This is mainly because about 70% of OC patients are already at an advanced stage once diagnosed and have lost the opportunity for radical surgery, so the 5-year survival rate is only 30% (STEWART et al., 2019). Therefore, from a long-term perspective, the prognosis of patients always depends on early diagnosis and prevention of recurrence in OC. Although the diagnosis of OC has been developed in recent decades, the prediction of diagnosis and prognosis in OC patients is still unsatisfactory. The recognized risk factors for OC include genetic risk, obesity, age, and the use of perineal talcum powder (PENNINKILAMPI and Eslick, 2018; LHEUREUX et al., 2019). But these factors are not yet considered a good source of help in predicting the prognosis of patients. Therefore, it is urgent to establish new biomarker model for OC diagnosis and prognosis prediction.

Tumor infiltrating immune cells (TIICs) are composed of a variety of cells in the tumor microenvironment, such as T cells, macrophages, neutrophils, and stromal cells (CHEW et al., 2012). Immunotherapy has a certain effect on OC, and the antitumor effect of infiltrating T lymphocytes in OC has already been observed (ZAMARIN, 2019). Recent studies have confirmed that the infiltration of immune-related cells and the abnormal expression of certain genes in these cells may be related to the occurrence and development of tumors, so these factors can also be used to predict the prognosis of OC (BACI et al., 2020; JIANG et al., 2020; NOWAK and KLINK, 2020). Similarly, stromal cells in tumors are also involved in tumor growth and drug resistance regulation, such as tumor-associated fibroblasts (YEUNG et al., 2016; De NOLA et al., 2019). The analysis and evaluation of immune cells and stromal cells can help us get a deeper understanding of the relationship between the tumor microenvironment (Corn et al., 2020) and prognosis of OC patients and help develop a reliable prognostic and predictive model.

Ferroptosis was proposed by DIXON in 2012 (DIXON et al., 2012). It is a new kind of programmed cell death that occurs after ferrous ions catalyze the formation of lipid peroxides. More and more evidences show that ferroptosis plays an important regulatory role in the occurrence and development of liver cancer (ZHANG et al., 2019), gastric cancer (LEE et al., 2020), and OC (CARBONE and MELINO, 2019; LIN and CHI, 2020; WANG et al., 2021). It can not only instruct the research of antitumor drugs but can also be used for OC biomarker screening. More importantly, ferroptosis-mediated iron ions, amino acid, reactive oxygen species, and lipid metabolism are closely related to the tumor immune microenvironment (Friedmann Angeli et al., 2019). At present, there is no report about the prognostic evaluation of the immune-stromal score combined with ferroptosis in OC patients, which has potential research value. In this article, we downloaded samples of OC patients from the public data sets TCGA and GEO. After standardizing the data, we constructed a prediction model of the immune-stromal score combined with ferroptosis-related genes through the TCGA training set and verified it in the TCGA training set and two validation sets of GEO. In addition, we analyzed the immune matrix infiltration of OC, ferroptosis gene co-expression network, and potential pathways. Compared with previous studies, this study has better credibility and more comprehensive data.

## Materials and Methods

### Data Source and Pre-processing

The gene expression data of the OC patients were downloaded from the TCGA and Gene Expression Omnibus (GEO) databases. The GEO datasets should fulfill the criteria that expression profiles were detected by array or high-throughput sequencing and contained corresponding clinical information, including age, histologic type, and overall survival (OS), at least. We obtained 375 samples in total with gene expression profiles from the TCGA OV dataset. In GEO datasets we collected GSE18520 which contained 53 OC samples and GSE32062 which contained 260 samples. OS refers to the time interval from the date of the patient’s first diagnosis to death. Among these datasets, we applied the TCGA OC dataset as the training set and the GEO datasets (GSE18520 and GSE32062) as validation sets. Affymetrix Human Genome U133 Plus 2.0 Array GPL570 served as the microarray platform for the GEO datasets, and the coding genes of patients with missing values were excluded. According to the annotation file provided by the platform, we annotated the microarray probe set to the gene name one by one. Then, log2 (Affy RMA) was used to unify the gene expression values. For genes containing several probes, the average value represented the expression value of the gene. For quality control and standardization during the data analysis, we used R package limma.

### The Analysis of Tumor-Infiltrating Immune Cells in Ovarian Cancer

First, the immune infiltration of TCGA patients was evaluated. R package “CIBERSORT” was applied to acquire the standardized abundance index of immune cells. Then, we used the LM22 gene signature and CIBERSORT algorithm to define 22 immune cells sensitively and specifically. The immune cells included the B cell family (naïve B cells, memory B cells, and plasma cells), T cell family (CD8^+^ T cells and naïve CD4^+^ T cells), M0 macrophages, M1 macrophages, resting NK cells, activated NK cells, resting dendritic cells, activated dendritic cells, resting mast cells, and monocytes). CIBERSORT is a deconvolution algorithm based on support vector regression and non-negative matrix factorization. We downloaded the reference gene expression value standard file LM22 corresponding to various immune cell types and called the corresponding package R script to calculate the proportion of different immune cells. Among them, LM22 is an expression matrix containing 547 genes, which serves as a standard control for distinguishing the proportion of immune cells. The main components of normal cells in tumor tissues are stromal cells and immune cells. These cells interfere with tumor signals in mechanism research and help promote tumor immune escape. By using expression data and R package “ESTIMATE”, the ESTIMATE algorithm could estimate the abundance of stromal cells and immune cells in tumors and speculate the ESTIMATE scores.

### Ferroptosis-Related Gene Acquisition

We collected 275 FRGs from the ferroptosis-related dataset FerrDb while removing the excess genes (http://www.zhounan.org/ferrdb). Regarding the 275 FRGs, we extracted a total of 244 genes which coexisted in TCGA-OV, GSE18520, and GSE32062 datasets. Then, the 244 genes were applied for the construction of a prognostic model.

### The Construction of the E-FRG Score Model

Through the “glmnet” package in R, we applied the least absolute shrinkage and selection operator (LASSO) regression analysis to determine the best weighting coefficient of FRGs in predicting OC prognosis. LASSO is an improvement of the least squares analysis. The core is to use penalty terms and regularization methods for statistical modeling and suppress overfitting. The best value of the penalty coefficient λ with the smallest partiality deviation was determined by running the lambda-min test, which gives the smallest cross-validation error. Therefore, the following formula was applied to speculate the E-FRGs score of each sample: E-FRGs score = ∑expgenei*βi. Expgenei represents the immune score, stromal score, and expression of the genes and βi represents the optimal coefficient for each factor included.

### Validation of the Estimate and Ferroptosis-Related Genes-Score Model

We applied the TCGA OC cohort as a training set for evaluating ESTIMATE and ferroptosis-related gene (E-FRG) score models, which contained 375 samples with integral gene expression profiles and necessary patient information. Then, we applied the GSE18520 and GSE32062 datasets to verify the predictive effect of the E-FRG score of OC. According to the critical value in TCGA, OC patients were divided into higher or lower E-FRG score groups. We choose the median as the critical value and use the median to verify the robustness of the model in the GSE18520 and GSE32062 datasets. Then we used Kaplan–Meier analysis to predict OS of OC patients while employing univariate and multivariate Cox regression analyses to determine independent prognostic factors between genes and other scores in E-FRGs in the training set. The receiver operating characteristic (ROC) curve was used to verify the preciseness and predictive ability of E-FRGs. The area under the curve (AUC) values of each risk model were calculated to determine the optimal risk signature. When the maximum AUC value was reached, the calculation procedure was terminated. The predictive ability of the risk signature for 1-/3-/5-year OS was assessed using the “survivalROC” R package. All patients were then categorized into the high-risk and low-risk groups based on the cutoff value identified in the training set. Kaplan–Meier (K-M) survival curves along with the log-rank test were used to identify differences in OS between the two groups using the R packages “survival” and “survminer”.

### The Analysis of Coexpression Gene Network

The “WGCNA” software package in R was used and weighted coexpression gene network analysis on FRGs in TCGA OC was performed. The main functions of WGCNA include clustering analysis of genes and calculating the association between FRGs and phenotypes. First, the correlation coefficients were calculated between the genes and determined gene modules. Then, a coexpression gene network was constructed, and the association between clinical features and gene modules was determined. Then, we used the integrated capability in WGCNA software to set the soft threshold power β to 10. FRGs were classified into four modules which illustrated the analogous expression modes on the basis of the hybrid dynamic cutting tree. We applied the cluster dendrograms to show the consequence of gene merging and classification. Finally, through Pearson’s correlation, the correlation between different modular genes and clinical characteristics has been evaluated.

### Clinical Tissues and Western Blotting Assays

We randomly collected a dozen human OC fresh tissues from patients undergoing surgery after obtaining their consent at The Second Xiangya Hospital of Central South University. Then, we analyzed the condition of these patients after receiving cisplatin treatment to select five specimens from cisplatin-sensitive patients (S1-S5) and five specimens from cisplatin-resistant patients (R1-R5). The experiment was approved by the Human Ethics Committee of The Second Xiangya Hospital of Central South University. Then, cancer cell lines in 6-cm dishes and tissues were lysed using RIPA buffer to obtain protein samples. Then, the samples were centrifuged at 12,000 g for 5 min at 4°C in a 1.5-ml tube, and the cell extract was transferred to a new tube. Then, 5*sodium dodecyl sulfate (SDS)–loading buffer was added and heated for 5 min at 95°C. The electrophoresis conditions were 120 V and 50 min. After electrophoresis, a polyvinylidene difluoride (PVDF) membrane was used for protein transfer at conditions of 400 mA at 45 min. The PVDF membrane was blocked using 5% skim milk diluted with Tris-buffered saline Tween 20 (TBST). The membrane was incubated with the following primary antibodies SLC7A5 (1:1,000, #13752-1-AP, Proteintech, United States), ACSL4 (1:2000, # 22401-1-AP, Proteintech, United States), XCT (1:2000, #26864-1-AP, Proteintech, United States), GPX4 (1:2 000, #14432-1-AP, Proteintech, United States), ALOX5 (1:2000, # 10021-1-Ig, Proteintech, United States), STEAP3 (1:4,000, # 17186-1-AP, Proteintech, United States), ZFP36(1:1,000, # 12737-1-AP, Proteintech, United States), GABARAPL1 (1:2000, #11010-1-AP, Proteintech, United States), NRAS (1:3,000, #10724-1-AP, Proteintech, United States) and beta actin (1:1,000, #20536-1-AP, Proteintech). After overnight incubation, the membrane was labeled using horseradish peroxide (HRP)–conjugated secondary antibody. Then, we washed the PVDF membrane and applied the electrochemiluminescence (ECL) luminescent solution to develop the pictures.

### Cell Culture and Cell Viability

OC cell lines, namely, OVCAR3 and SKOV3 were obtained from Sciencell (California, United States) and maintained in RPMI 1640 medium supplemented with 10% fetal bovine serum. To establish CDDP-resistant cells, namely, OVCAR3/CDDP and SKOV3/CDDP, the cells were treated with cisplatin (#HY-17394, MedChemExpress, China) in a stepwise manner from 0.2 to 2 μg/ml. Then, the cells were transferred to a cisplatin-free medium for three days before the beginning of the experiments to reduce the influence of cisplatin. The drugs and their concentrations involved in cell viability, GSH, and MDA assays included cisplatin (2.2 or 6.9 μg/ml), erastin (10 μM, #S7242, Selleck, United States), RSL3 (0.1 μM, #S8155, Selleck, United States) and acetylcysteine (N-acetyl-l-cysteine, NAC) (1 mM, #S1623, Selleck, United States), while their treatment time was 24 h. The cells were seeded on 96-well plates at a density of 1.5 × 10^4^cells/ml and cultured 24 h before drug treatment. Then, cell viability was detected by using the CellTiter Blue^®^ reagent (Promega, G8082, United States). A water bath maintained at 37°C was employed to remove the reagent, and 20 μl/well of the CellTiter Blue^®^ Reagent was added. Then, the plates were incubated in standard cell culture conditions for 2 h, and the fluorescence intensity was measured at 560/590 nm.

### Glutathione Assay and Lipid Peroxidation Assay

The cells were treated with 5% 5-sulfosalicylic acid (SSA) solution. Then, the GSH level was determined by using the reduced GSH Assay kit (#K464-100, BioVision) according to the manufacturer’s protocol. The results were normalized to total protein concentration for each sample. Lipid peroxidation could be detected by the combination of malondialdehyde (MDA) with thiobarbituric acid (TBA) through the MDA assay kit (#MAK085, Sigma). The cells were treated in MDA lysis buffer and then centrifugated at 13,000 g for 3 min. The absorbance wavelength of products formed by MDA and TBA was measured at 532 nm.

### Comparison of Enriched Oncogenic Pathways

We used Kyoto Encyclopedia of Genes and Genomes (KEGG) analysis to figure out potential signaling pathways of FRGs while using gene ontology (GO) enrichment analysis to explain the functions of FRGs. And the “clusterProfiler” software package was used for visualization. We conducted GO analyses at a standard of *p-*value < 0.01 and FDR<0.05. In addition, we performed Gene Set Enrichment Analysis (GSEA) on FRGs at a standard of *p-*value < 0.05. In order to further explain the potential relationship between the pathways involved in FRGs in OC and immune cells, the Gene Set Variation Analysis (GEVA) method was applied to obtain the pathway-score matrix. Pearson’s correlation analysis was applied between the GSVA and CIBERSORT matrices.

### Statistical Analysis

All statistical analyses were conducted using R and Rstudio software (R version 4.0.3). The Wilcoxon test was used to judge the difference of two groups in single gene expression. Pearson’s correlation test was used to determine the correlation between immune cell ratios and pathway scores. According to the risk score, the sample was divided into two groups, and the Kaplan–Meier survival curve was established using the log-rank test. An ROC curve was constructed to evaluate the sensitivity and specificity of the risk score of the E-FRG survival prediction model. All statistical tests were two-sided tests, and a *p-*value <0.05 was considered statistically significant.

## Results

### The Relationship Between the Tumor-Infiltrating Immune Cells and Clinical Characteristics of Ovarian Cancer Patients

**FIGURE 1:**
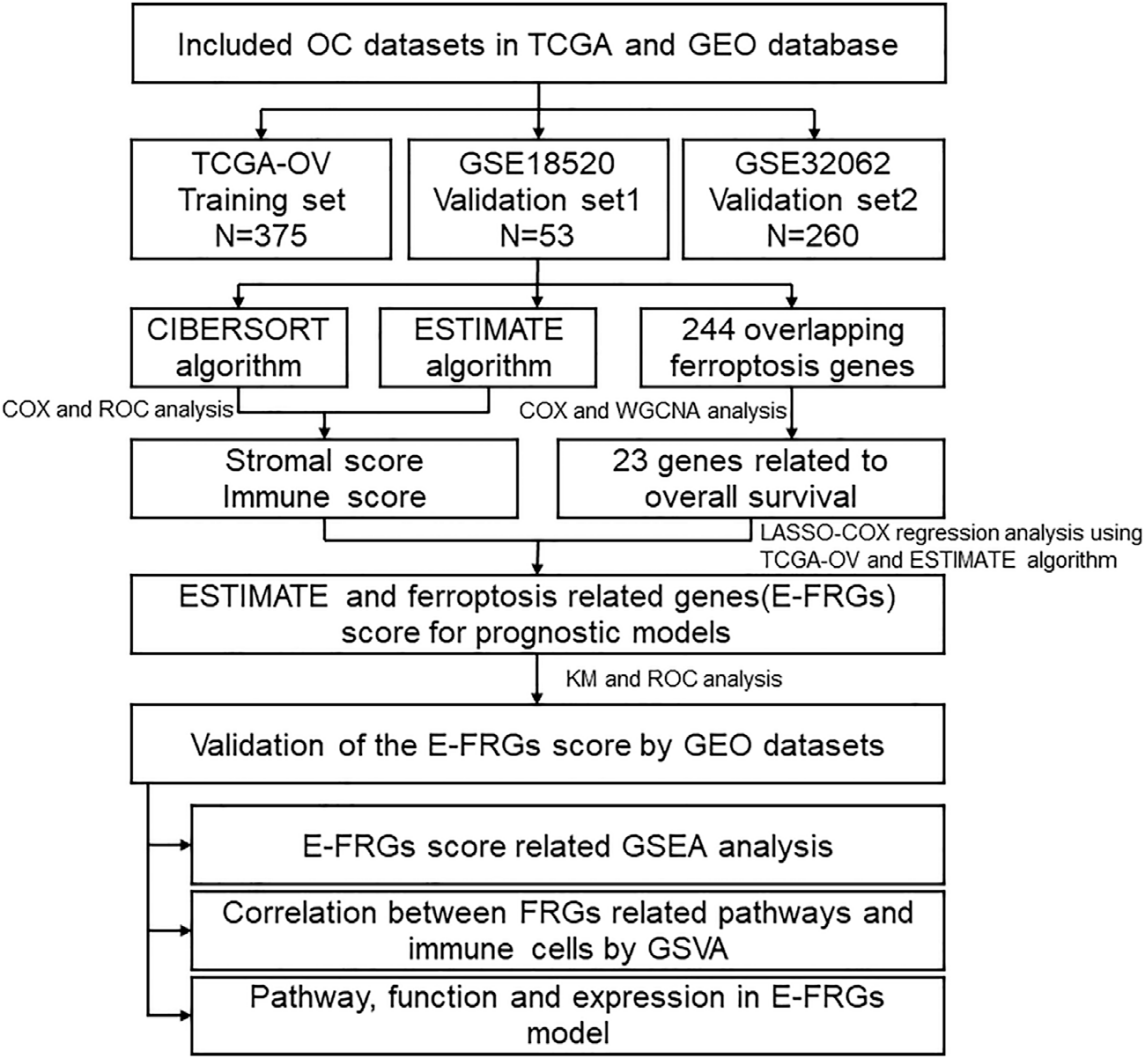
Overall flowchart. First, use ESTIMATE and CIBERSORT were used to analyze the relationship with the prognosis of OC patients. Furthermore, the ESTIMATE scores and ferroptosis-related gene expression were combined to construct the E-FRG model. Finally, the possible regulatory pathway was analyzed based on the E-FRGS model.

**FIGURE 2:**
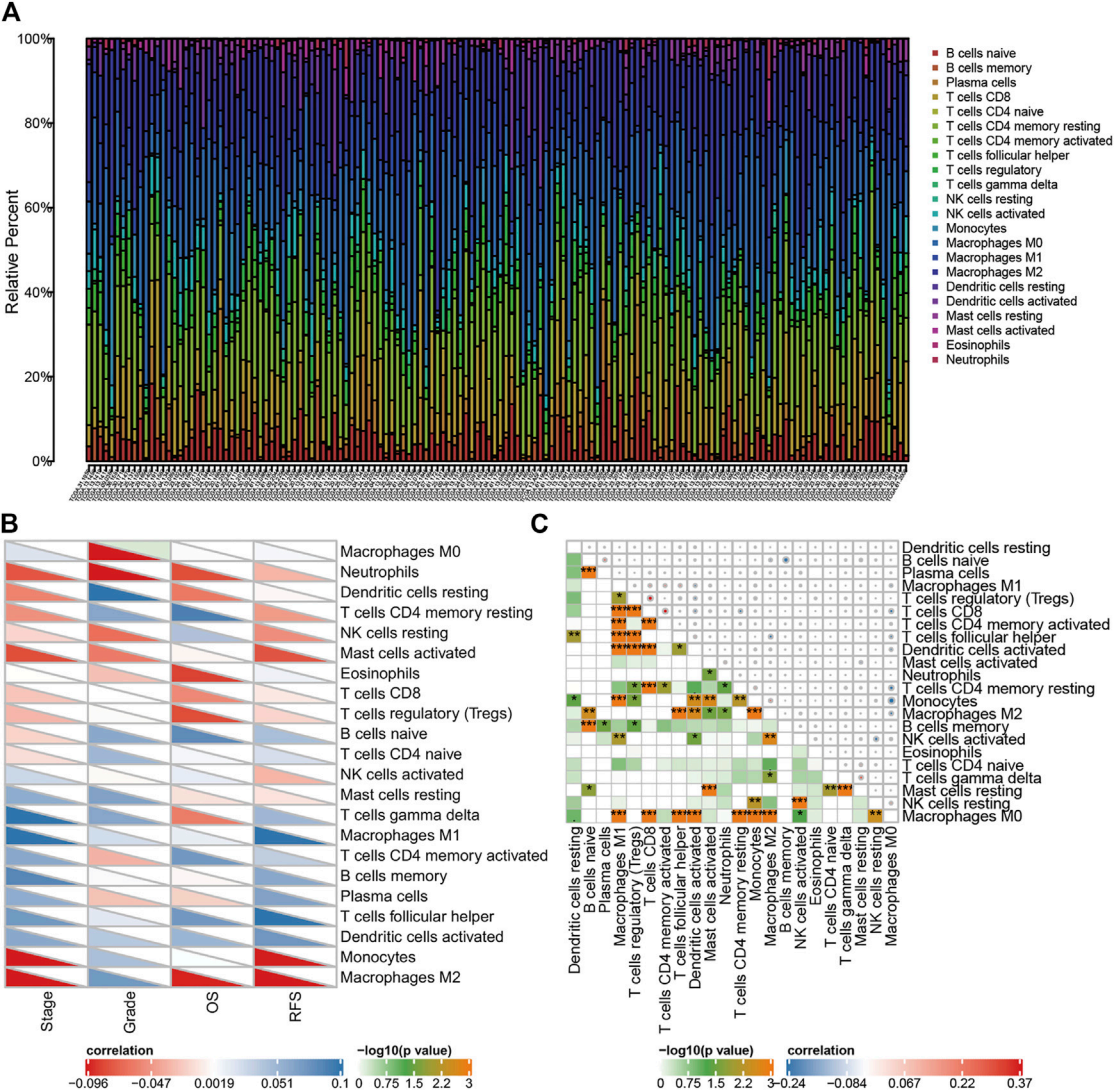
Distribution of tumor immune cells in OC patients, and the relationship between immune infiltration cells and clinical features. **(A)** Proportion of 22 kinds of TIICs in TCGA OC samples was shown in the bar plot. Horizontal axis represents different patient samples and the vertical axis represents the percentage of TIIC. **(B)** Correlation of 22 TIICs with clinicopathologic-grade characteristics was calculated by Pearson’s correlation test. **(C)** Correlation among 22 kinds of TIICs. Color in the lower left corner represents the size of the *p-*value, and the size and color of the circle in the upper right corner represent Pearson’s correlation coefficient. *p < 0.05, **p < 0.01, and ***p < 0.001.

The flow diagram of the procedures of our study is illustrated in Figure 1. In this study, three datasets were enrolled which contained a total of 688 OC samples. The tumor microenvironment cells modulate the antitumor response and play important roles in predicting clinical prognosis and treatment effect. Using the CIBERSORT algorithm, we summarized the 22 subpopulations of TIICs of the 375 OC samples in the training set, including the B cell family (naïve B cells, memory B cells, and plasma cells), T cell family (CD8^+^ T cells and naïve CD4^+^ T cells), M0 macrophages, M1 macrophages, resting NK cells, activated NK cells, resting dendritic cells, activated dendritic cells, resting mast cells, and monocytes. The distribution of immune cells was significantly different among individuals, which depicted a comprehensive individual immune characteristic in OC (Figure 2A). Then, the relationship between each subtype of TIICs and clinical characteristics in the training set was analyzed, including stage, grade, RFS, and OS. We found that M0 macrophages were identified to be associated with grade (*p*=.069), which was elevated while the TNM stage increased (Figure 2B). Then, the correlation analysis showed a comprehensive landscape of TIIC interactions in OC. The results showed that some tumor immune cells had a high connection with others, including CD8 T cells and M0 macrophages, CD8 T cells and resting dendritic cells, naïve B cells and memory B cells, follicular helper T cells and M0 macrophages, activated dendritic cells and M0 macrophages, gamma delta T cells and resting mast cells, and M1 macrophages and monocytes. (Figure 2C)

To construct a prognostic model, the univariable Cox regression of all 22 TIICs in the training set was constructed to find independent prognostic factors for OS. We defined plasma cells, M1 macrophages, monocytes, follicular helper T cells, M2 macrophages, and activated mast cells as risky prognostic factors, whose *p-*values are 0.013, 0.030, 0.031, 0.001, 0.009, and 0.003 respectively. Then, we applied multivariable Cox proportional hazard regression analysis with the two subtypes of TIICs. As a result, the included two cells showed a poor predictive effect on the prognosis of OC patients. So, we determined to introduce other factors to establish a prognostic model for OC (Supplementary Table S1).

### Correlation With the Estimation of Tumor-Infiltrating Cells and Prognosis

**FIGURE 3:**
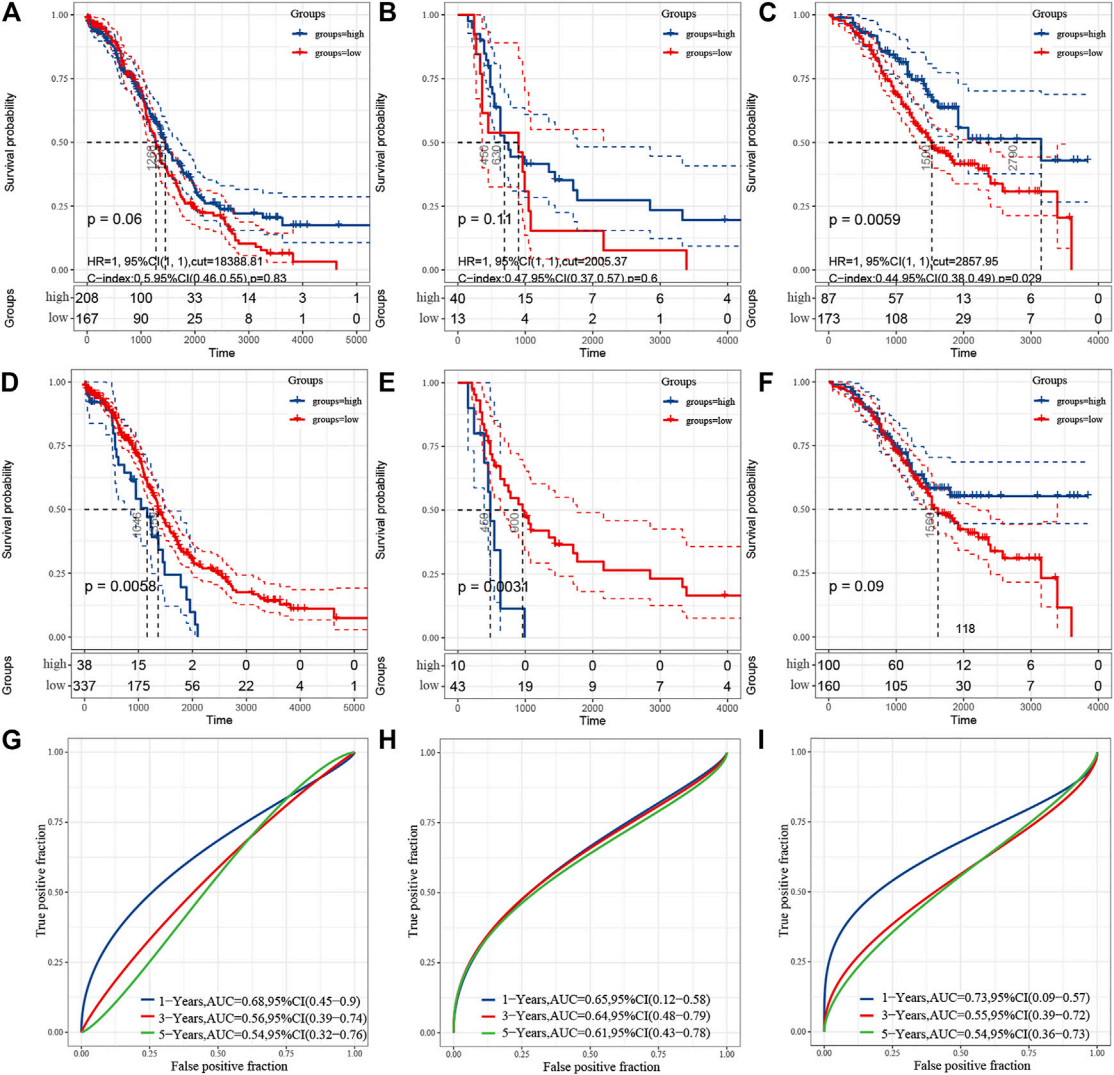
Correlation with the estimation of tumor-infiltrating cells and prognosis. **(A–C)** Kaplan–Meier survival analysis showing association between immune score and OS. **(D–F)** Kaplan–Meier survival analysis showing association between stromal score and OS. **A** and **D** represent TCGA, **B** and **E** represent GSE18520, C and F represent GSE32062. G–I, ROC curve for measuring the predictive value of immune score or stromal score for OS in GSE18520. **(G)** represents the immune score. **(H)** represents the stromal score. **(I)** represents the ESTIMATEscore.

To figure out the degree of tumor immune cell infiltration in samples, the ESTIMATE algorithm was applied using the gene expression profiles in OC. The TCGA, GSE18520, and GSE32062 datasets were applied. The ESTIMATE algorithm could generate three scores: immune score, stromal score, and ESTIMATE score. These scores were tested by Kaplan–Meier survival analysis and ROC curves to assess their prediction capability. The immune score was related to the survival in TCGA(*p* = 0.06) (Figure 3A), GSE18520 (*p* = 0.11) (Figure 3B) and GSE32062 (*p*=.0059) datasets (Figure 3C). The stromal score was significantly related to the survival in TCGA(*p*=.0058) (Figure 3D) and GSE18520 (*p* = 0.0031) (Figure 3E), but not related to GSE32062 (*p*=.09) (Figure 3F). The ESTIMATE score was significantly related to the survival in all three datasets, whose *p-*values were 0.031, 0.023, and 0.011 respectively. Because the ESTIMATE score is the sum of the aforementioned two scores, we display it in Supplementary Figure S1. The stromal score achieved the highest area under the curve of ROC (AUC) in 1-, 3-, and 5-year ROC analysis in GSE18520 (Figures 3G–I), while the ESTIMATE score and immune score acquired the highest AUC in 1-year ROC analysis in it. These results indicate that the prognostic prediction capability of the immune score was not effective enough, so we needed more factors to establish a predictive model.

### The Analyses of Survival-Related Ferroptosis-Related Genes in Ovarian Cancer

**FIGURE 4:**
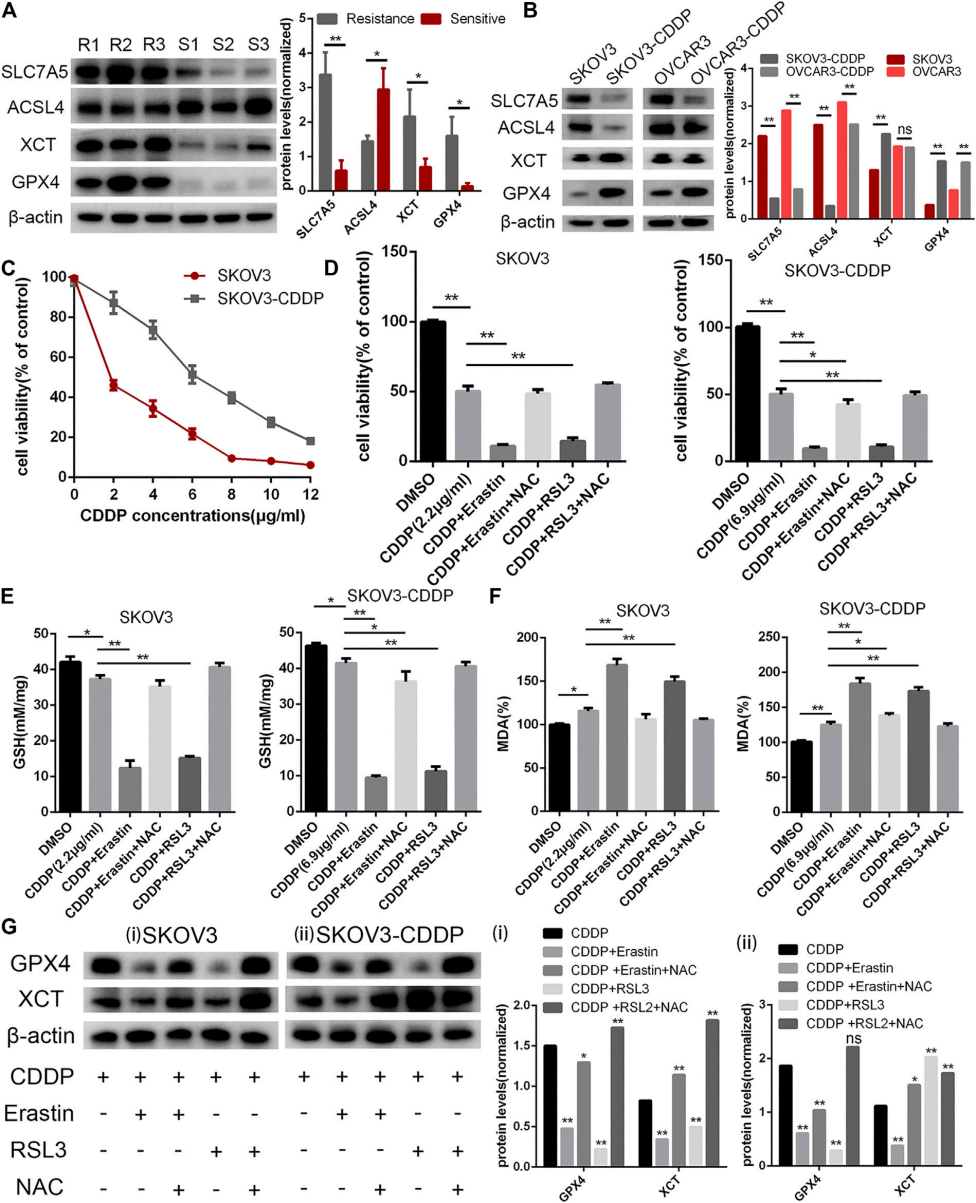
Analyses of survival-related FRGs in OC. **(A)** Ferroptosis pivot genes, namely, SLC7A5, ACSL4, XCT, GPX4, and β-actin protein expression in platinum-resistant tissues (R1-R3) and platinum-sensitive tissues (S1-S3). **(B)** SLC7A5, ACSL4, XCT, GPX4, and β-actin protein expression in ovarian cell lines (OVCAR3 and SKOV3) and platinum-resistant OC cell lines (OVCAR3-CDDP and SKOV3-CDDP). **(C)** Cell viability assays under cisplatin treatment; SKOV3/CDDP showed higher cisplatin tolerance, representing the resistance phenotype. Cells were treated with cisplatin (2.2 μg/ml for SKOV3 and 6.9 μg/ml for SKOV3-CDDP) in the absence or presence of erastin (10 μM), RSL3 (0.1 μM), and NAC (1 mM) for 24 h and cell viability. **(D)** Glutathione (GSH) **(E)** and lipid peroxidation (MDA) assays **(F)** were performed. **(G)** XCT, GPX4, and β-actin protein expression in SKOV3 **(i)** and SKOV3-CDDP **(ii)** under the abovementioned treatment. Error bars represent the standard deviation (s.d.) of triplicate measurements. *p < 0.05 and **p < 0.01.

**FIGURE 5:**
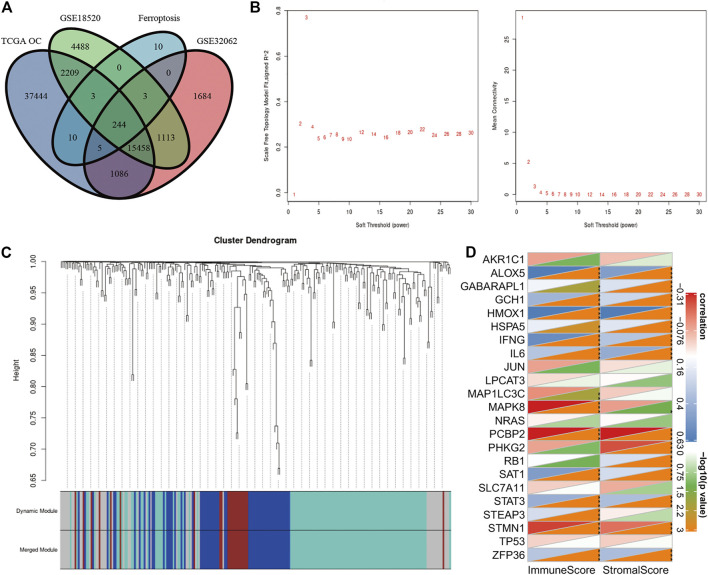
**(A)** Venn diagrams are used to show the overlapping FRGs in FerrDb, TC GA OV, GSE18520, and GSE32062. **(B)** Scale-free fit index for soft-thresholding powers. Ordinate on the left represents the square of the correlation coefficient between log (k) and log [p (k)] in the FRG network. Ordinate on the right represents the mean value of the adjacent function of all genes in the corresponding gene module. **(C)** Cluster dendrogram of ferroptosis-related genes. Each branch represents a gene, and the colors below represent specific co-expression modules. **(D)** Pearson’s correlation analysis between 23 ferroptosis-related genes and the results calculated by ESTIMATE. *p < 0.05, **p < 0.01, and ***p < 0.001.

Though there have been some studies about ferroptosis in OC in recent years, the relationship between ferroptosis, cisplatin resistance, and prognosis of OC has not been clear (LIN and CHI, 2020). We collected three clinical tissues from platinum-sensitive patients (S1-S3) and three tissues from platinum-resistant patients (R1-R3) to analyze the protein level of ferroptosis pivot proteins. Then, we found that proteins which inhibited ferroptosis (SLC7A5, XCT, and GPX4) were elevated in platinum-resistant tissues, while ACSL4 which inhibited ferroptosis was elevated in platinum-sensitive tissues (Figure 4A). The protein expression had the same tendency in OC cell lines and corresponding platinum-resistant cell lines (OVCAR3/OVCAR3-CDDP and SKOV3/SKOV3-CDDP) (Figure 4B). In order to further verify the relationship between ferroptosis and platinum resistance in OC cells, we tested the antitumor effect of the ferroptosis agonist (erastin or RSL3) combined with cisplatin. Eastin and RSL3 can inhibit the activity of XCT and GPX4, respectively, to induce ferroptosis, while acetylcysteine (N-acetyl-l-cysteine, NAC) could inhibit ferroptosis through a mitochondrial-dependent pathway. The IC50 values of cisplatin were 2.2 and 6.9 μg/ml for SKOV3 and SKOV3/CDDP cells, respectively, which was applied in the following experiments (Figure 4C). The cell viability assays demonstrated that ferroptosis agonists, namely, erastin and RSL3 could both reinforce the cytoxic effect of cisplatin in SKOV3 and SKOV3-CDDP, while NAC could rescue these lethal combinations (Figure 4D). We then examined the accumulation of the end product of lipid oxidation, namely, malondialdehyde (MDA) in the treated cells, which was a marker for oxidative stress and ferroptosis. The content of GSH was also quantified. It showed that erastin and RSL3 could induce ferroptosis by GSH depletion and lipid peroxidation in SKOV3 and SKOV3-CDDP–treated with cisplatin (Figures 4E,F). Western blotting assays demonstrated the different protein expression in GPX4 and XCT after the treatment of ferroptosis agonists, namely, NAC and cisplatin, which proved the aforementioned points (Figure 4G). These data together suggested that ferroptosis agonists could induce ferroptosis to strengthen the lethal effect of the IC50 concentration of cisplatin in OC cells, which reminded us that ferroptosis may participate in recovering platinum resistance and had potential efficacy in cooperating with cisplatin in OC cells. The gene symbols of the three datasets were integrated with 244 ferroptosis-related genes (FRGs) obtained from the FerrDb database (http://www.zhounan.org/ferrdb) for further analysis (Figure 5A). We used WGCNA to analyze gene expression data of overlapping FRGs in the TCGA dataset to establish a co-expression gene network. The soft-thresholding power was defined on the basis of scale-free R2 (R2 = 0.95). Through the power and average linkage hierarchical clustering, we figured out four modules (Figures 5B,C). The co-expression gene network has been illustrated in eigengenes. Subsequently, the correlation analysis of each eigengene with clinical characteristics, including OS, grade, stage, and RFS, was conducted. As presented in Supplementary Figure S2, the gray module was positively correlated with RFS (*p* = 0.01) in OC patients. The blue module was negatively correlated with OS (*p* = 0.02), and the turquoise module was negatively correlated with OS (*p* = 0.006), stage (p = 1e-04) and RFS (*p* = 0.01). The gray module contained 44 FRGs and the turquoise contained 115 FRGs. It is reported that the ferroptosis-related genes are involved in tumor stromal invasion and immune process (STOCKWELL and JIANG, 2019), but it is not clear how the correlation works in OC. We first selected 23 genes related to survival from the data of the aforementioned 244 gene sets and performed Pearson’s correlation analysis with the results calculated by the ESTIMATE algorithm. The results showed that 15 genes each were significantly related to immune scores and stromal scores in the ESTIMATE algorithm. This suggests that the ferroptosis-related genes (FRGs) are closely related but not completely collinear with ESTIMATE (Figure 5D). The combination of these two indexes may obtain a more accurate prognostic model.

### Construction and Validation of the Estimate and Ferroptosis-Related Gene Model by Integrating Estimate and Ferroptosis-Related Genes

**FIGURE 6:**
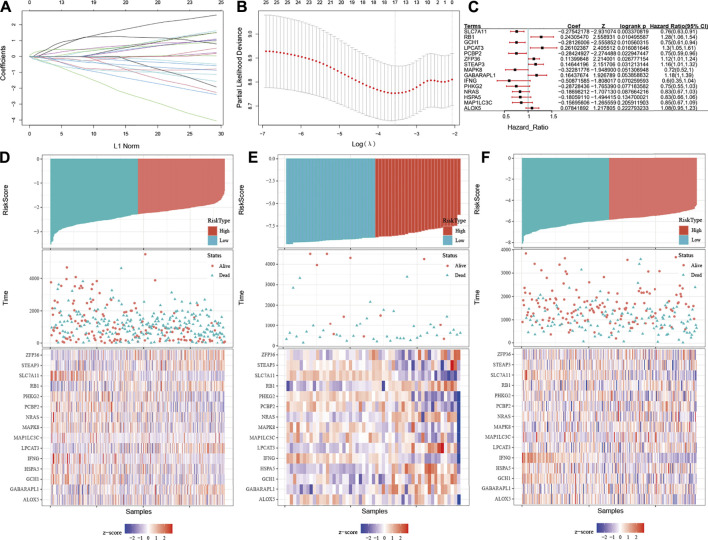
Construction of the E-FRG model by integrating ESTIMATE and FRGs. **(A)** LASSO coefficient profiles of the 23 FRGs, immune score, and stromal score. Coefficient profile plot was produced versus the log (λ). **(B)** Tuning parameter (λ) selection in the LASSO-Cox regression model was performed using 10-fold cross-validation via the 1 standard error for the minimum criteria. Black vertical lines were plotted at the optimal λ based on the minimum criteria and 1 standard error for the minimum criteria. **(C)** Forest plot showing *p-*values of FRGs in the model indicated by the color scale presented on the side. Horizontal bars represent 95% confidence intervals. **(D–F)** Distribution between the high E-FRG score group and low E-FRG score group in the TCGA dataset **(D)**, GSE18520 **(E)**, and GSE32062 **(F)**, respectively.

Since the ESTIMATE algorithm cannot precisely distinguish patients with OC with high-risk well, we decided to introduce FRGs into the prognostic risk prediction model for OC. Recently reported FRGs are associated with platinum resistance and poor prognosis in OC, while ferroptosis could affect the activation and function of immune cells (Friedmann Angeli et al., 2019; CHAN et al., 2020; WANG et al., 2021; MA et al., 2021). It could be inferred that the integration of the immune and FRGs may be able to assess the condition of OC patients and form an effective prognostic predictive risk model. Univariable Cox proportional hazard regression analysis was applied with initial screening of 244 FRGs, and 23 FRGs were finally identified based on the log-rank test. Next, the LASSO–Cox regression model was conducted integrating the ESTIMATE algorithm and these FRGs (E-FRGs) (Figures 6A,B). Finally, the immune score, stromal score, and 15 FRGs were determined in the E-FRG model, which included solute carrier family seven member 11 (SLC7A11), RB transcriptional corepressor 1 (RB1), GTP cyclohydrolase 1 (GCH1), lysophosphatidylcholine acyltransferase 3 (LPCAT3), poly (rC)-binding protein 2 (PCBP2), zinc finger RNA-binding protein (ZFP36), STEAP3 metalloreductase (STEAP3), mitogen-activated protein kinase 8 (MAPK8), GABA type A receptor–associated protein like 1 (GABARAPL1), interferon gamma (IFNG), phosphorylase kinase catalytic subunit gamma 2 (PHKG2), NRAS, hot shock protein A5 (HSPA5), microtubule-associated protein one light chain three gamma (MAP1LC3C), polyunsaturated fatty acid 5-lipoxygenase (ALOX5), ESTIMATE score, and stromal score (Figure 6C). In the E-FRG model, the risk score (E-FRG score) was generated using the following formula:

E-FRG score=(0.019×ALOX5) + (0.108×GABARAPL1) + (-0.122×GCH1) + (-0.054×HSPA5) + (-0.326×IFNG) + (0.105×LPCAT3) + (-0.061×MAP1LC3C) + (-0.181×MAPK8) + (-0.145×NRAS) + (-0.240×PCBP2) + (-0.104×PHKG2) + (0.142×RB1) + (-0.281×SLC7A11) + (0.011×STEAP3) + (0.041×ZFP36) + (-1.19E-06×stromal score) + (-9.91E-06-06×immune score).

**FIGURE 7:**
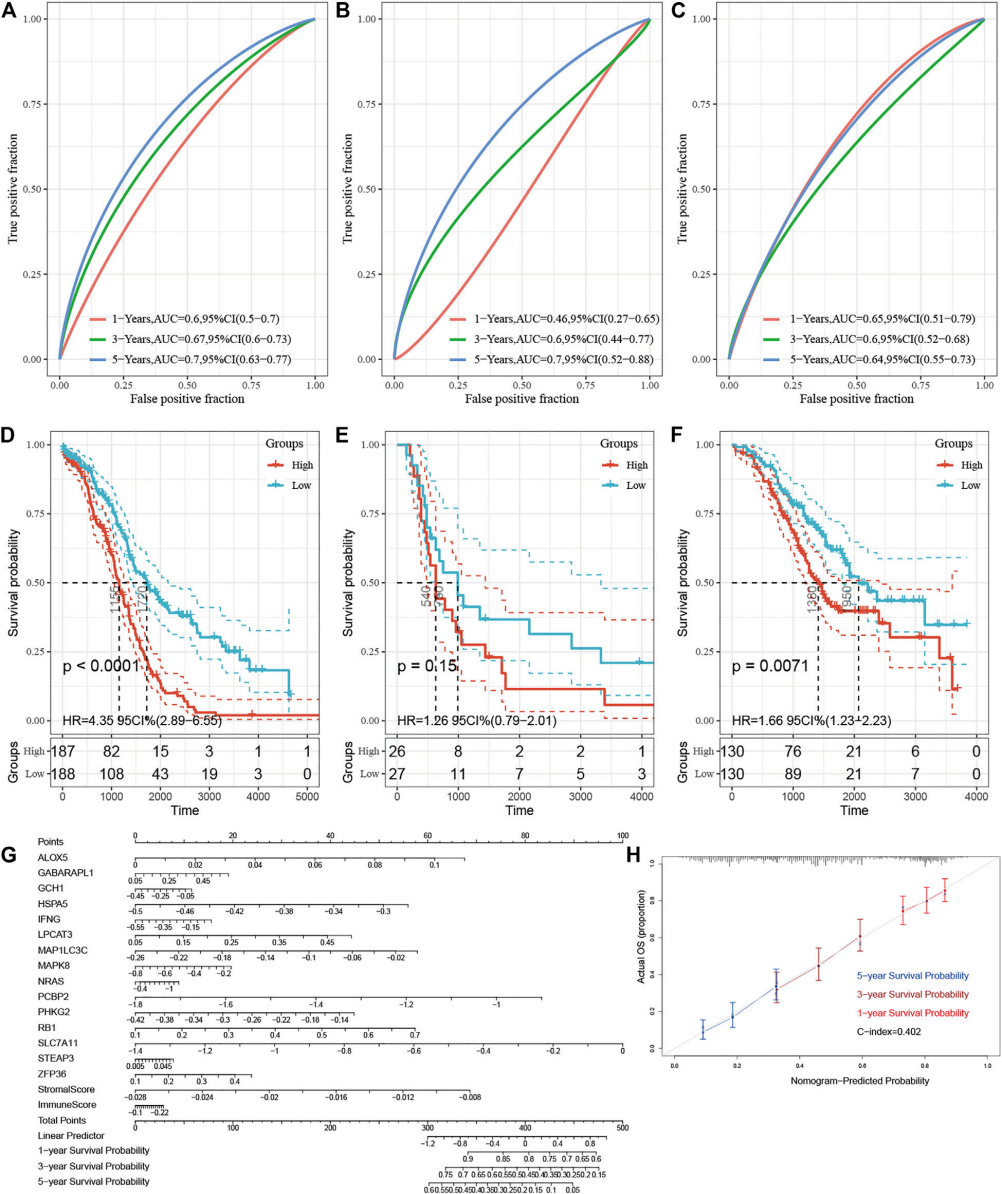
Validation of the E-FRG model and development of a nomogram for improving the model. **(A–C)** Area under the curve of the ROC curve is used to visually indicate the predictive power of E-FRG scores for 1-, 3-, and 5-year OS of OC patients in the TCGA dataset **(A)**, GSE18520 **(B)**, and GSE32062 **(C)**, respectively. **(D–F)** Kaplan–Meier curves for 1-, 3-, and 5-year OS with low and high E-FRG scores in the TCGA dataset **(D**), GSE18520 **(E)**, and GSE32062 **(F)**, respectively. **(G)** Nomogram for predicting the 1-, 3-, and 5-year OS probabilities of OC patients. The total score is composed of 17 scoring features. Each score of the nomogram corresponds to the genes or ESTIMATE scores in the E-FRG model. **(H)** Calibration of the nomogram for predicting the probability of survival at 1-, 3-, and 5-year in TCGA (C-dex = 0.402).

Then the E-FRG score was calculated for the patients enrolled in the TCGA dataset with the corresponding score. The optimal cutoff value was determined by the median value of -2.26, -8.75, and -5.81 in TCGA, GSE18520, and GSE32062, respectively (Figures 6D–F). Subsequently, the included OC patients could be classified into high-risk and low-risk groups on the basis of the median value. The Kaplan–Meier curve analysis showed that the E-FRG model could distinguish OC patients with good or bad prognosis. The high–E-FRG group manifested a shorter OS than the OS of low–E-FRGs group in the TCGA dataset (*p* < 0.0001, Figure 7D). In GSE18520, patients with high risk showed shorter OS with a marginally significant *p-*value of 0.15 (Figure 7E). In GSE32062, the OS was significantly shorter in the patients with high risk (*p* = 0.0071, Figure 7F).

Time-dependent ROC analysis showed that AUC of E-FRG scores for the prediction of 1-, 3-, and 5-year OS in the training set were 0.60, 0.67, and 0.70, respectively (Figure 7A). In GSE18520, the AUC value of the E-FRG scores for predicting 1-, 3-, and 5-year OS were 0.46,0.60, and 0.70, respectively (Figure 7B), while those in GSE32062 were 0.65,0.60, and 0.64, respectively (Figure 7C). It is worth noting that the E-FRG score showed better predictive effect than other potential prognostic markers on the basis of time-dependent ROC analysis (Supplementary Figure S3).

### The Development of a Nomogram for Improving the Estimate and Ferroptosis-Related Gene Model

As a regression model illustrated in images, the nomogram is widely applied in the diagnosis of tumors and the prognostic analysis. The nomogram and calibration curve were applied in our study in order to illustrate the E-FRG model more vividly and improve the practicality of this model (Figures 7G,H). The nomogram included more than 17 traits, including the 15 FRGs, immune score, and stromal score. The score of each characteristic was determined by the scale on the top. The sum of the scores of the 17 traits was defined as the final score. We could estimate the prognosis of 1-, 3-, and 5-year OS for OC patients by the perpendicular line from the total point axis to the two-outcome axis.

### Defining Ferroptosis-Related Gene-Related Pathways and Immune Cells by Gene Set Enrichment Analysis and GSVA Analysis

**FIGURE 8:**
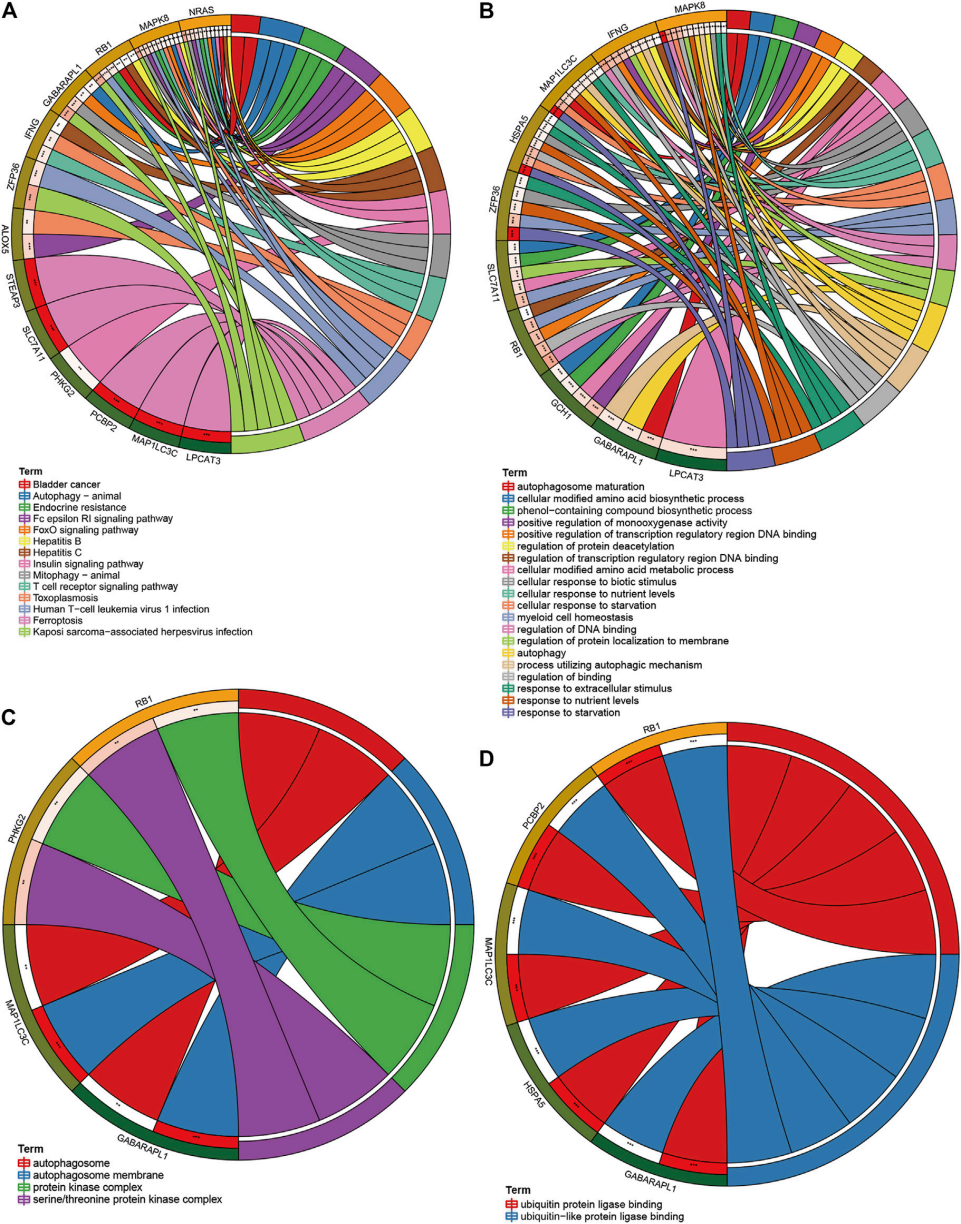
Defining FRG-related pathways and immune cells by GSEA and GSVA analysis. **(A,B)** GSEA analysis between the low and high E-FRG score groups. **(C)** GSVA analysis on FRGs in the TCGA training set which provided 22 FRG-related pathways. **(D)** Correlation of 22 TIICs with 22 FRG-related pathways. Pearson’s coefficient was used for significance test. *p < 0.05, **p < 0.01, and ***p < 0.001.

The enrichment analysis of ferroptosis is helpful to explain why the E-FRG score can better predict the prognosis of OC patients. We first used E-FRG scores to perform KEGG–GSEA analysis and selected eight pathways related to E-FRG scores. Among them, adipocytokine, glycosaminoglycan biosynthesis chondroitin sulfate, focal adhesion, and ECM receptor interaction signaling pathway are positively correlated with E-FRG scores (Figure 8A) and cell cycle, one carbon pool by folate, RNA degradation, and protein export are negatively correlated (Figure 8B).

In addition, ferroptosis is closely related to the regulation and activation of immune cells (HASCHKA et al., 2020). Exploring the immune-related FRG pathway is conducive to the discovery of drugs that have the ability to activate ferroptosis and immunity at the same time. We first performed GSVA analysis on FRGs (Figure 8C) and calculated the correlation between the GSVA and CIBERSORT scores (Figure 8D). The results showed that autophagy, leishmania infection, and the PPAR signaling pathway were positively correlated with follicular helper T cells, mast cells, and neutrophils and negatively correlated with monocytes and eosinophils.

### Encyclopedia of Genes and Genomes, Gene Ontology Analysis, and Expression of Genes in the Estimate and Ferroptosis-Related Gene Model

**FIGURE 9:**
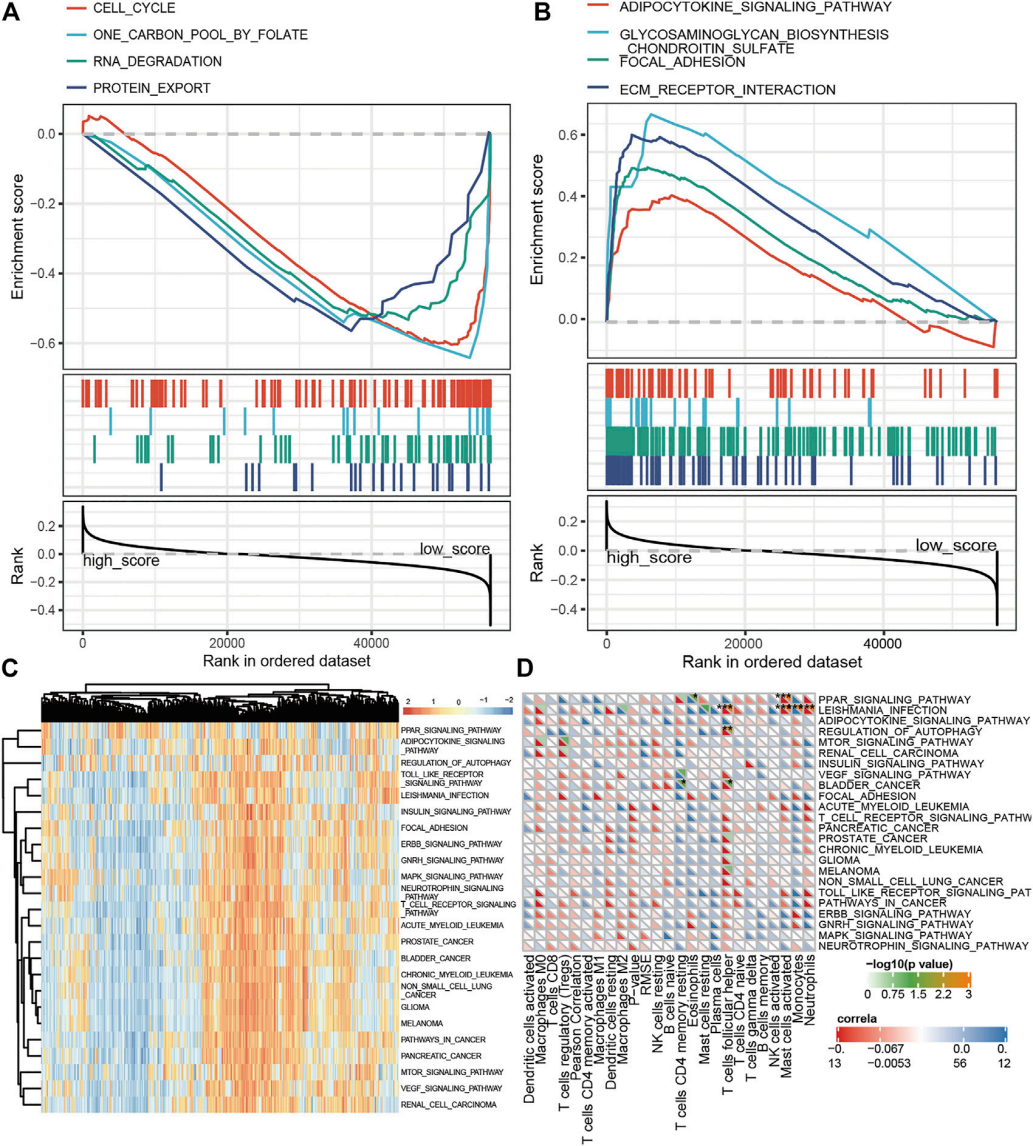
KEGG, GO analysis of FRGs in the E-FRG model. **(A)** KEGG analysis showing main pathways related to ferroptosis. (B–D) GO analysis showing significant GO terms related to FRGs. **(B)** FRGs related to biological processes. **(C)** FRGs related to cell components. **(D)** FRGs related to molecular function.

After the overall enrichment analysis of FRG, we performed KEGG and GO analysis on 15 genes in the E-FRG model and also collected their RNA and protein expression. In the results of KEGG analysis, autophagy, endocrine resistance, and immune pathways are the main pathways related to ferroptosis (Figure 9A). Ten genes are enriched in GO biological processes, and these processes are mainly involved in autophagy, amino acid metabolism, various oxidative stress reactions, and subsequent DNA damage (Figure 9B). Cell component analysis suggests that four genes are involved in the autophagosome and protein kinase complex (Figure 9C). The results of GO molecular function suggest that five genes, such as RB1 and HSPA5, are enriched in the ubiquitination process (Figure 9D).

**FIGURE 10:**
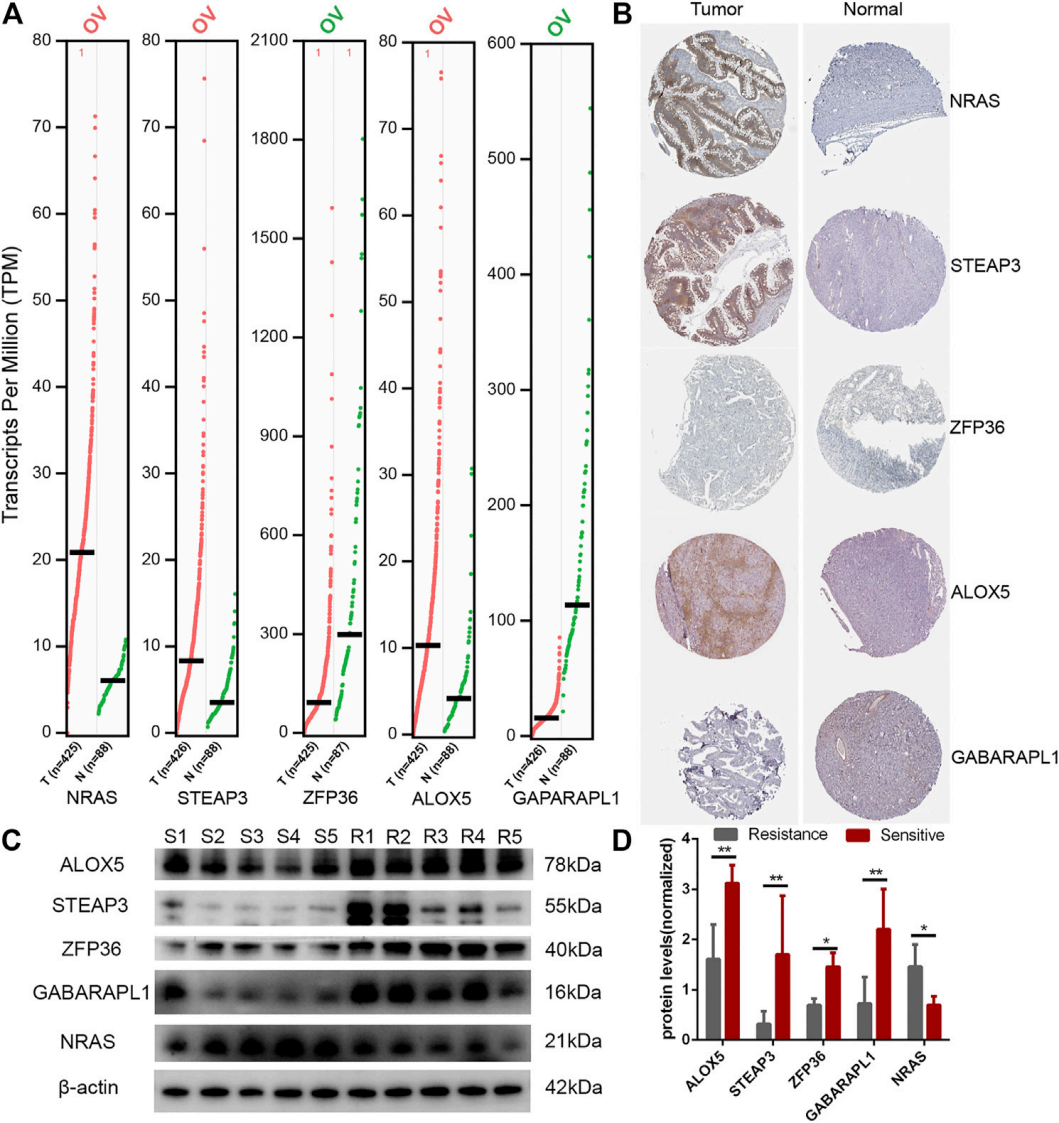
Expression of FRGs in the E-FRG model. **(A)** GEPIA database showing the expression of TOP5 different genes in OC tissues compared with normal tissues. **(B)** HPA database showing the expression of TOP5 different genes in OC tissues compared with normal tissues. **(C)** Protein expression of TOP5 genes in the E-FRG model (ALOX5, STEAP3, ZFP36, GABARAPL1, and NRAS) and β-actin in platinum-resistant tissues (R1-R5) and platinum-sensitive tissues (S1-S5). **(D)** Error bars represent the standard deviation (s.d.) of quintuplicate measurements. *p < 0.05 and **p < 0.01.

Finally, we showed TOP5 different expression genes in the E-FRG model. Compared with normal tissues, NRAS, STEAP3, and ALOX5 are highly expressed in OC and ZFP36 and GABARAPL1 are lowly expressed (Figure 10A). In the HPA database, NRAS, STEAP3, and ALOX5 proteins are significantly expressed in tumor tissues, but the difference in the expression of ZFP36 and GABARAPL1 proteins is not obvious (Figure 10B). Then, we analyzed the expression of TOP5 genes in five clinical tissues from platinum-sensitive patients (S1-S5) and five tissues from platinum-resistant patients (R1-R5). It illustrated that the genes negatively correlated with E-FRG model (NRAS) were elevated in platinum-sensitive tissues, while genes positively correlated with the E-FRG model (ALOX5, STEAP3, ZFP36, and GABARAPL1) were elevated in platinum-resistant tissues (Figures 10 C,D).

## Discussion

OC is the most difficult malignant tumor to treat among common gynecological tumors (BRAY et al., 2018). Immunotherapy and targeted therapy have improved the prognosis of patients to a certain extent. However, the current prognosis of patients based on immunotherapy is still unpredictable, so it is necessary to explore models that can accurately predict the prognosis of OC patients. More and more studies have shown that immune cells or stromal cell infiltration are related to the survival of OC patients (LI et al., 2021). Ferroptosis is a new type of programmed cell death, and many ferroptosis-related genes are involved in immune regulation. These genes have been found to have expression changes after targeted therapies in tumors, so they may be used to predict the prognosis of OC patients (YE et al., 2020). Research studies on ferroptosis or immunity have also been reported. In 2021, Yang et al. reported a prognostic model consisting of nine ferroptosis-related genes by multi COX regression analysis, but only 60 ferroptosis-related genes were included and the immune–stromal score was not involved in the study (YANG et al., 2021). Here, we first evaluated the immune cell infiltration of OC and the evaluation effect of the ESTIMATE score on the prognosis of OC. Based on collecting ferroptosis-related genes as much as possible, the E-FRG model combining ESTIMATE scores and FRGs was developed and verified by the LASSO-COX method. The E-FRG model used to identify high-risk patients has good discrimination in both training and validation datasets.

In this study, the constructed prognostic model consisted of two ESTIMATE scores and 15 genes related to ferroptosis. These genes regulate ferroptosis through a variety of metabolic pathways. STEAP3 is an endosomal ferrireductase which functions as an iron and copper transporter in erythroid cells. STEAP3 maintains the homeostasis of iron ions in cells by reducing Fe3+ to Fe2+, thus participating in the regulation of ferroptosis in bone marrow injury (WILKINSON et al., 2019). SLC7A11, also called XCT, is a signature protein of ferroptosis and participates in intracellular and extracellular cysteine transport to regulate the production of glutathione (GSH) (KOPPULA et al., 2020). PCBP2 is the main poly (rC)-binding protein in the cell. It was previously thought to act as an adaptor between the mitochondrial antiviral signaling protein (MAVS) and E3 ubiquitin ligase, triggering the ubiquitination and degradation of MAVS. It was also thought to participate in the transduction of mitochondrial antiviral signals (YANATORI et al., 2016). NRAS regulates the activity of GTPase and is one of the most common targets of targeted drugs, as well as the upstream target of drugs, such as sorafenib. Many studies believed that RAS gene activation is an important prerequisite for tumor cell ferroptosis (SU et al., 2020). RB1 is a key regulator of cell division. The active form of RB1 binds to E2F transcription factor 1 and inhibits the transcriptional activity of it, leading to cell cycle arrest. Louandre found in sorafenib-treated liver cancer cells in which the downregulation of RB1 promoted the occurrence of ferroptosis (LOUANDRE et al., 2015). MAPK8 is an important member of the serine/threonine protein kinase family, responsible for regulating the proliferation and apoptosis of glioblastoma cells (XU et al., 2018). In ferroptosis, the MAPK pathway is often studied as a downstream of RAS (NGUYEN et al., 2020).

MAP1LC3C, also known as LC3C, is a key protein in the autophagy pathway and the ubiquitin-like modification of heterophagy (CHO et al., 2020). This model also includes GABARAPL1, which also belongs to the LC3 family of autophagy and is responsible for the formation of autophagic vesicles (PARK et al., 2016). Ferritinophagy is a specific autophagosome formed by ferritin and autophagy. This study suggests that MAP1LC3C may be involved in this process, but it has not been further confirmed by studies. ZFP36 is a C2H2 zinc finger protein, which destabilizes the transcription containing the AU-rich element (ARE) by removing or deadenylating the poly (A) tail of RNA, thus attenuating protein synthesis. Since ZFP36 is a key factor regulating ROS homeostasis, it has been confirmed that ZFP36 prevents ferroptosis in hepatic stellate cells through the autophagy signaling pathway (ZHANG et al., 2020). PHKG2 is the catalytic subunit of PHK which participates in glycogen decomposition and hormone regulation by activating glycogen phosphorylase. PHKG2 regulates the effectiveness of iron ions on lipoxygenase and then drives ferroptosis through the peroxidation of polyunsaturated fatty acids (PUFA) at the diallyl position (YANG et al., 2016). LPCAT3 is a phosphatidylcholinease that participates in and maintains the homeostasis of phospholipid metabolism and regulates tumorigenesis. Consistent with the study of Yang et al., the model we constructed also believes that LPCAT3 is an important iron death gene for predicting the prognosis of OC (YANG et al., 2021).

ALOX5 belongs to the lipoxygenase family, which is rich in iron and is responsible for lipid oxidation. ALOX5 catalyzes the peroxidation of PUFA, which is the limit of the leukotriene biosynthesis and ferroptosis process. Ferroptosis can further lead to positive feedback amplification of itself by reducing ALOX5-mediated inflammation (LIU et al., 2015). IFNG is an important macrophage activator which has a regulatory effect on the phenotypic transformation of tumor-associated macrophages and can resist the growth of pancreatic tumors (HUANG et al., 2020). Studies have confirmed that IFNG released by cytotoxic T cells downregulates the expression of glutamate transport systems (SLC3A2 and XCT), thereby promoting lipid peroxidation and iron drop in cancer cells, which explains the close relationship between immunity and ferroptosis (WANG et al., 2019). Endoplasmic reticulum stress is also believed to interact with ferroptosis. HSPA5 is a kind of heat shock protein. As a downstream of UPR, it may inhibit ferroptosis (ZHU et al., 2017). GCH1 induces lipid remodeling by regulating the synthesis of tetrahydrobiopterin/dihydrobiopterin, thereby selectively preventing phospholipid consumption and inhibiting ferroptosis (KRAFT et al., 2020).

In summary, we constructed a model that can effectively predict the prognosis of OC patients based on ESTIMATE scores and ferroptosis-related genes. Our E-FRG model provides new perspectives for the improvement of individualized management of OC patients. In addition, this study found that the level of the E-FRG score is related to the degree of infiltration of immune cells and stromal cells. These ferroptosis-related genes also interact with signal pathways, such as autophagy and immunity, suggesting that these genes may be key nodes in the crosstalk of pathways.

## Data Availability

The data that support the findings of this study are openly available in the Cancer Genome Atlas (TCGA) database for TCGA-OV at https://www.cancer.gov/t-cga, the Gene Expression Omnibus (GEO) database for GSE32062 at https://www.ncbi.nlm.nih.gov/geo/query/acc.cgi?acc=GSE32062, for GSE73293 at https://www.ncbi.nlm.nih.gov/geo/query/acc.cgi?acc=GSE73293.
