# Supplementary material for: Comprehensive Analysis of the Tumor Microenvironment and Ferroptosis-Related Genes Predict Prognosis with Ovarian Cancer
Source: Front Genet. 2021 Nov 15;12:774400. doi: 10.3389/fgene.2021.774400 (PMC8634641; doi:10.3389/fgene.2021.774400)
Supplement: Supplementary file 1 [file DataSheet1.docx]

**Supplementary Table 1** The univariable Cox proportional hazards regression of all 22 TIICs for OS in TCGA dataset

| Tag | P value | HR | Low 95%CI | High 95%CI |
| --- | --- | --- | --- | --- |
| B cells naive | 0.967484 | 0.942026 | 0.053332 | 16.63929 |
| B cells memory | 0.593806 | 0.304334 | 0.003843 | 24.09992 |
| Plasma cells | 0.013268 | 0.001839 | 1.26E-05 | 0.268821 |
| T cells CD8 | 0.851096 | 0.829093 | 0.117152 | 5.867568 |
| T cells CD4 naive | 0.279244 | 6.36E+61 | 7.13E-51 | 5.67E+173 |
| T cells CD4 memory resting | 0.168703 | 3.414029 | 0.594151 | 19.61724 |
| T cells CD4 memory activated | 0.084775 | 0.007336 | 2.74E-05 | 1.961886 |
| T cells follicular helper | 0.029606 | 0.006473 | 6.90E-05 | 0.607149 |
| T cells regulatory (Tregs) | 0.651392 | 0.32533 | 0.002494 | 42.44125 |
| T cells gamma delta | 0.384301 | 0.026017 | 7.00E-06 | 96.6988 |
| NK cells resting | 0.468525 | 48.39359 | 0.001347 | 1738564 |
| NK cells activated | 0.668383 | 2.147378 | 0.065056 | 70.88098 |
| Monocytes | 0.030538 | 13.81895 | 1.279596 | 149.2372 |
| Macrophages M0 | 0.845299 | 0.90954 | 0.350907 | 2.357501 |
| Macrophages M1 | 0.001113 | 0.010402 | 0.000668 | 0.161879 |
| Macrophages M2 | 0.009261 | 7.765627 | 1.658496 | 36.36123 |
| Dendritic cells resting | 0.136716 | 87.66633 | 0.242102 | 31744.36 |
| Dendritic cells activated | 0.417258 | 0.435025 | 0.058224 | 3.250304 |
| Mast cells resting | 0.333452 | 0.247031 | 0.014527 | 4.20068 |
| Mast cells activated | 0.002927 | 485.4293 | 8.254028 | 28548.68 |
| Eosinophils | 0.605462 | 5.205456 | 0.009955 | 2721.933 |
| Neutrophils | 0.073425 | 69823.57 | 0.3472 | 1.4E+10 |


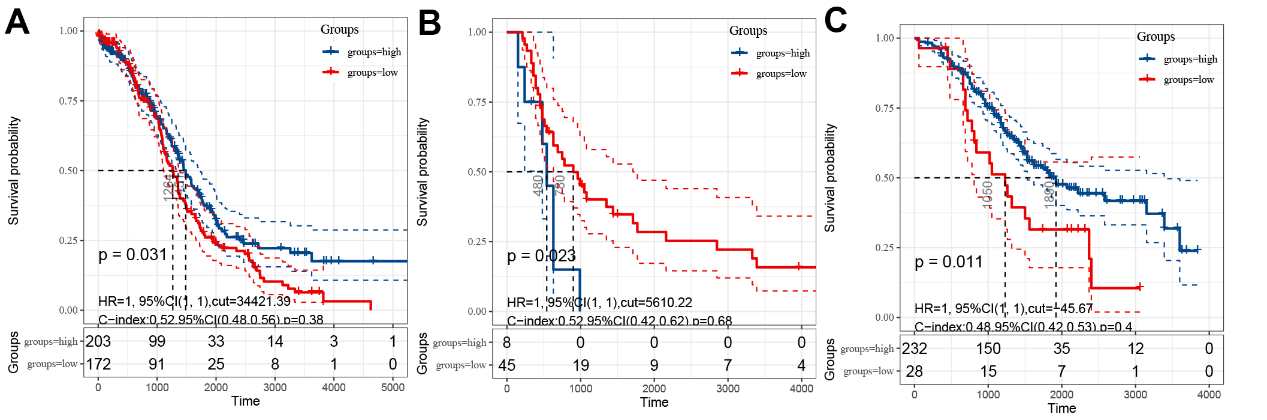


**Supplementary Figure 1** Kaplan–Meier survival analysis showing that high ESTIMATEscore were associated with better OS in OC patients. ***A,*** TCGA dataset. ***B,*** GSE18520 dataset. ***C,*** GSE32962 dataset.


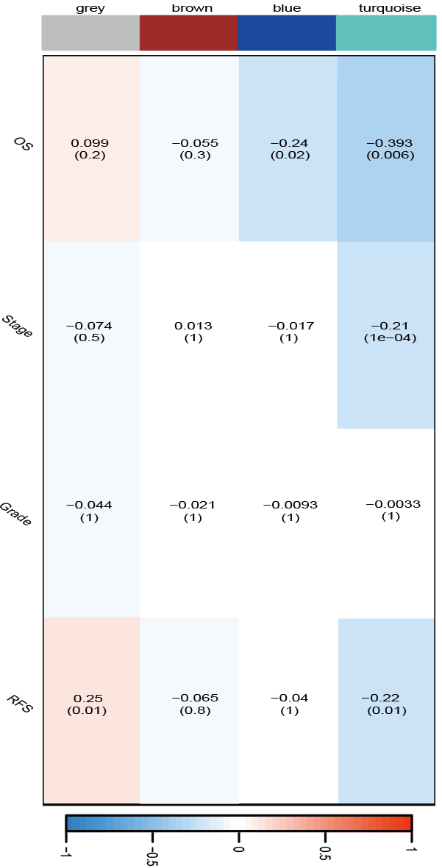


**Supplementary Figure 2** The relationship between co-expressed gene modules and clinical characteristics. The data in each grid represents the correlation coefficient between gene modules and clinical features.


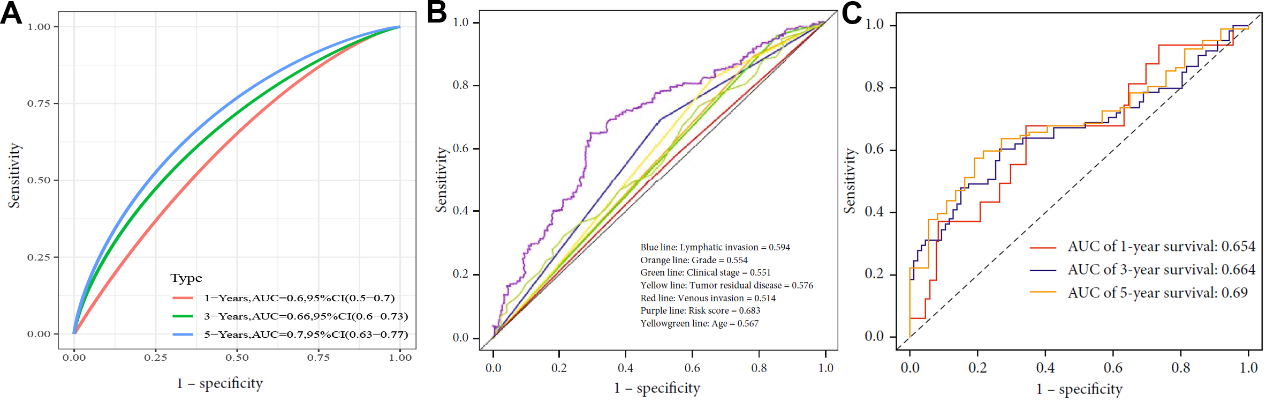


**Supplementary Figure 3** The AUC of the E-FRGs score for 1-, 3-, and 5-year overall survival predictions compared with other studies. ***A,*** Results of the E-FRGs score. ***B,*** Results from the article of Yang et al., adapted with permission from (1), copyright 2021, Hindawi Limited. ***C,*** Results from the article of An et al., adapted with permission from (2), copyright 2020, Hindawi Limited.

**References:**

1. YANG L, TIAN S, CHEN Y, MIAO C, ZHAO Y, WANG R, et al. Ferroptosis-Related Gene Model to Predict Overall Survival of Ovarian Carcinoma. J ONCOL (2021); 2021: 6687391.

2. AN Y, YANG Q. Development and Validation of an Immune-Related Prognostic Signature for Ovarian Cancer Based on Weighted Gene Coexpression Network Analysis. BIOMED RES INT (2020); 2020: 7594098.
